# We Can Quit2 (WCQ2): a community-based intervention on smoking cessation for women living in disadvantaged areas of Ireland—study protocol for a pilot cluster randomised controlled trial

**DOI:** 10.1186/s40814-019-0511-9

**Published:** 2019-11-23

**Authors:** Catherine Hayes, Aurelia Ciblis, Catherine Darker, Nadine Dougall, Joanne Vance, Nicola O’Connell, Fiona Dobbie, Kirsty Loudon, Emma Burke, Declan Devane, Linda Bauld

**Affiliations:** 1 Public Health & Primary Care, Institute of Population Health, School of Medicine, Trinity College Dublin, Russell Centre, Tallaght Cross, Dublin, D24 DH 74 Ireland; 2 School of Health and Social Care, Edinburgh Napier University, Sighthill Court, Edinburgh, EH11 4BN UK; 3 Irish Cancer Society, 43/45 Northumberland Road, Ballsbridge, Dublin 4, Ireland; 4 Usher Institute, College of Medicine and Veterinary Science, University of Edinburgh, Edinburgh, EH8 9AG UK; 5 Scotland, UK; 6 HRB Trials Methodology Research Network & School of Nursing & Midwifery, NUI Galway, Galway, Ireland

**Keywords:** Smoking cessation, Women and smoking, Community-based intervention, Social and health inequalities, Cluster randomised controlled trial, Feasibility study, Pilot trial, PRECIS-2, Process evaluation

## Abstract

**Background:**

Tobacco use is the leading cause of preventable death in Ireland with almost 6000 smokers dying each year from smoking-related diseases. The ‘We Can Quit2’ (WCQ2) study is a pilot pragmatic two-arm, parallel-group, cluster randomised trial that aims to explore the feasibility and acceptability of trial processes including recruitment and to estimate parameters to inform sample size estimates needed for an effectiveness trial. This future trial will assess the effectiveness of a community-based smoking cessation intervention for women living in disadvantaged areas on short- and medium-term cessation rates.

**Methods/design:**

Four matched pairs of districts (eight clusters) selected by area level of deprivation, geographical proximity, and eligibility for free medical services will be randomised to receive either WCQ (behavioural support + access to Nicotine Replacement Therapy (NRT)) delivered over 12 weeks by trained Community Facilitators (CFs) or to a form of usual care, a one-to-one smoking cessation service delivered by Smoking Cessation Officers from Ireland’s national health service, the Health Service Executive (HSE). Within each cluster, 24–25 women will be recruited (97 per arm; 194 in total) in 4 phases with consent obtained prior to cluster randomisation. The outcome measures will assess feasibility and acceptability of trial processes, including randomisation. Outcome data for a future definitive intervention (biochemically validated smoking abstinence) will be collected at end of programme (12 weeks) and at 6 months. WCQ2 has an embedded process evaluation using both qualitative and quantitative methods. This will be conducted (semi-structured client and CF interviews, intervention delivery checklist, and diary) to explore acceptability of trial processes, intervention fidelity, trial context, and implementation. Trial processes will be assessed against domains of the PRECIS-2 wheel to inform a future definitive trial design.

**Discussion:**

Data from this pilot trial will inform the design and sample size for a full cluster randomised trial to determine the effectiveness of an intervention tailored to disadvantaged women in improving smoking cessation rates. It will provide transferable learning on the systems and implementation strategies needed to support effective design of future pragmatic community-based trials which address health promotion interventions for women in disadvantaged communities.

**Trial registration:**

Concurrent to publication. Controlled trials ISRCTN74721694.

## Background

Tobacco use is the leading cause of preventable death globally. Almost 6000 smokers die annually in Ireland from smoking-related diseases [1]. The Tobacco Free Ireland Programme have set a target for Ireland to become tobacco free (i.e. with a smoking prevalence rate of less than 5%) by 2025 [2]. Although smoking rates have dropped by 1% per year since 2015, 20% of the population aged 15 years and over remained regular smokers in 2018 (men 22%; women 17%) [3]. The data confirm persistence of the health inequalities gap with higher rates in more disadvantaged than in more affluent areas (26% vs 16%) [3]. It is evident therefore that ‘business as usual’ is not sufficient to affect the step change required to reach the endgame [4].

Whereas lung cancer incidence rates in Irish males declined steadily during 1994 to 2015, female rates increased significantly and continue to increase [5]. More women in Ireland are now dying from lung cancer (19% of all cancer deaths) than breast cancer (17%) [6]. In socio-economically disadvantaged areas, lung cancer rates are higher in women than in men [5]. This pattern suggests that smoking impacts on health inequalities via gender in addition to socio-economic influences.

Research has also noted the cumulative effect of disadvantage on women’s smoking [7]. The intergenerational impacts of women smoking are particularly evident in disadvantaged communities, with increased likelihood of potentially preventable adverse outcomes perinatally, during infancy and childhood [8]. Measures to restrict impact of environmental tobacco smoke in the home are less likely to be implemented [9], and parental smoking in the home increases the child’s chances of becoming an adult smoker (via role modelling) which has direct and immediate impact on children’s health [10].

Previous studies have made the case that smokers from disadvantaged groups should be targeted for greater efforts to support smoking cessation, with more consideration of the role of gender in design of smoking prevention interventions. Such efforts need to focus on the social conditions that affect women’s lives as well as the individual level interventions including approaches to improve health behaviours [7].

The link between cancer and smoking among women in Ireland, and other developed countries, has been recognised by the World Health Organisation (WHO) through the Framework Convention on Tobacco Control [8]. The WHO has called for a gendered lens to be applied to tobacco control policies. Despite this, few countries have attempted to tailor smoking cessation services to meet the needs of women.

The national Health Service Executive (HSE) provides smoking cessation services which are not gender-based. This service is available to any Irish citizen. The universal components of this service are a National Quit service, which was launched in 2014 as an interactive smoking cessation support service delivered via phone, web, text, and social media. These elements may be supported by specialist smoking cessation services locally that provide face-to-face behavioural support either individually or group based (rarely) and access to Nicotine Replacement Therapy (NRT); however, this is resource dependent [9]. A map of the Irish smoking cessation services is available at the following link: https://quit.ie/I-Want-to-Quit/Support-Services/.

### WCQ

Arising from the above context and in a bid to design and deliver an intensive, face-to-face smoking cessation intervention tailored to women that is feasible in an Irish context, the Irish Cancer Society (ICS) in partnership with the National Women’s Council Ireland, and the Institute of Public Health in Ireland developed ‘We Can Quit’ (WCQ), a community-based smoking cessation programme for women from socially and economically disadvantaged communities.

The WCQ intervention was developed in accordance with the Medical Research Council guidance for developing complex interventions, identifying the evidence base, identifying/developing theory, and modelling process and outcomes [10]. It was developed based on evidence that delivery of a group programme to disadvantaged US women in their local communities by trained peers (sister-to-sister) [11, 12] using behavioural support and access to pharmacotherapy [13] resulted in higher than expected smoking cessation rates in this group of women. The intervention was adapted by the ICS and HSE for an Irish setting, using a community social-ecological model [14] and optimised in a prior feasibility study of 39 women which showed favourable retention and smoking cessation rates [15].

The core components of the WCQ intervention are as follows:

- An evidence-based smoking cessation behavioural support programme delivered in a group setting over 12 weeks with individual follow-up as required for women from disadvantaged communities
- Combination NRT (nicotine patches and an oral NRT product dispensed by community pharmacists free of charge)
- Delivery by trained community health workers to women in their community setting
- Inclusion of a relapse prevention element

Additional non-core components include the following:

- Signposting/additional community supports by local community health workers to participants who enrol in the programme
- Use of social marketing strategies to promote cultural change and promote cessation services

The principal differences with the standard HSE programme are the tailoring of WCQ to women in a group community setting, delivery by community lay personnel over 12 weeks as opposed to an average of 6–7 individual sessions, and NRT provided free of charge to all participants instead of a means-tested service within the HSE.

The 12-week programme was modified for this pilot cluster randomised controlled trial (RCT)—WCQ2, by revision of the intervention manual and standardisation of all questionnaires to include validated questions. This protocol describes the WCQ2 RCT which incorporates an embedded process evaluation.

### Aims

The overall aim of the study is to assess the feasibility of running a definitive randomised trial to determine whether a community-based smoking cessation complex intervention tailored to disadvantaged women affects their smoking cessation rates.

### Objectives

The primary objectives of the trial are as follows:

(i) To determine the feasibility and acceptability of trial processes including randomisation of districts, recruitment, and data collection in both intervention and control arms
(ii) To assess data quality and completion rates for the main outcome measures for a future definitive trial (DT) including biochemically validated abstinence from smoking (using salivary testing) at 12 weeks (end of programme) and 6 months (longer-term outcome)
(iii) To estimate the sample size and design for a future DT including an estimate of the intra-cluster correlation coefficient (ICC) to account for the effect of ‘clustering’ in design and analysis

The secondary objectives of the trial are as follows:

(i) To test the robustness of trial design with respect to context for delivery of the intervention, implementation processes, and key mechanisms of impact, from which to optimise design of a full effectiveness trial
(ii) To develop strategies to optimise recruitment and dissemination of findings to trial stakeholders to inform knowledge exchange and future research

The study protocol (version 3.0, 19 October 2018) has been developed in line with the Standard Protocol Items: Recommendations for Interventional Trials (SPIRIT) 2013 statement [16] (Additional file 1). Any changes to the protocol will be communicated to all investigators, submitted for ethical approval, and reflected in changes to the trial registry.

## Methods/design

This study is a pilot two-arm, parallel group, cluster randomised trial [17]. It was designed with assistance from the Irish Health Research Board Trials Methodology Research Unit (HRB-TMRN).

### Setting, recruitment, and informed consent

#### Cluster recruitment

Four matched pairs of urban and semi-rural districts (eight clusters) in Dublin and Cork will be identified in advance where local communities have expressed interest in participating via Local (Area) Advisory Groups (LAGs).

LAGs have been developed, with two in Dublin and two in Cork, through a community engagement process initiated and developed by the ICS in partnership with a company in the local community and are government funded. They consist of the ICS, the Local Development Company, community development projects, family resource centres, representatives from the HSE, local authorities, local pharmacies, and other relevant community groups, such as traveller, women, and youth organisations.

Their function is to establish support for community action on cancer prevention, including provision of community-based smoking cessation services for disadvantaged groups. In the WCQ2 study, their role will be to direct and deliver a local recruitment strategy. They will support the delivery of the research project in their designated areas.

Criteria for selection of the four matched pairs of districts by the LAGs will be similarity in terms of population size where we aim to include approximately 8,000 - 10,000 women per district, deprivation using the 2016 Pobal HP Deprivation Index [18], and feasibility such as availability of a one-to-one national smoking cessation service in the chosen areas. The selected LAGs will be asked to provide informed consent based on their understanding of randomisation of their district to either an intervention or a control arm.

### Participant recruitment

#### Eligibility criteria

Women will be invited to take part in the study if they are 18 or over, speak fluent English, self-report as daily smokers, indicate an interest in quitting, are deemed to live/work/reside within easy travel distance of trial catchment areas, are taking NRT, or have been prescribed bupropion/varenicline by the doctor at time of recruitment and women using e-cigarettes who are current smokers.

#### Exclusion criteria

The following participants will be excluded from the study: women who are pregnant or planning a pregnancy in the near future, women already enrolled in another smoking cessation study, those who cannot travel to location of programme delivery, women who use NRT/bupropion/varenicline/e-cigarettes who have not smoked cigarettes in the 7 days prior to recruitment, and women who do not have the capacity to give informed consent.

### Sample size

**Fig. 1:**
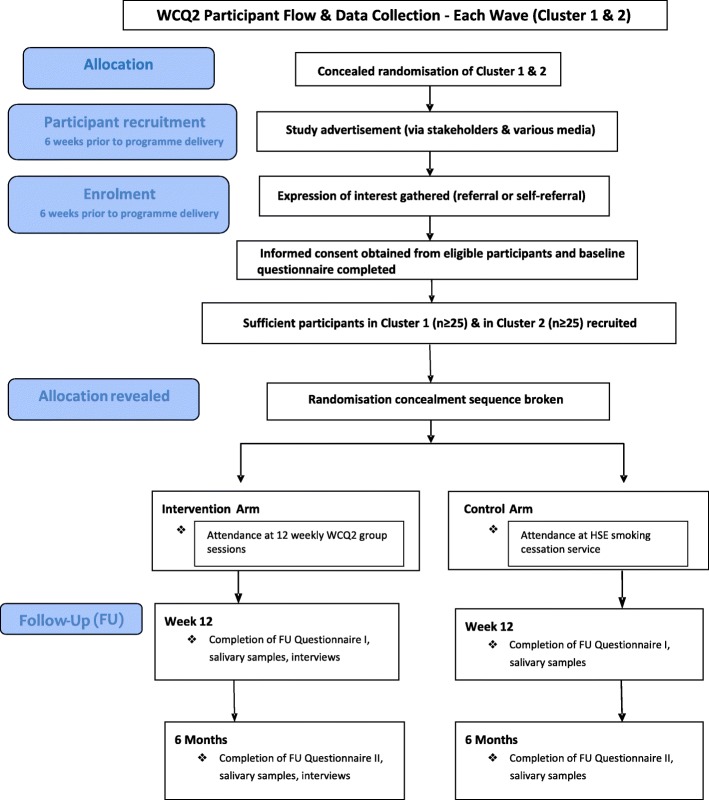
Trial schema and participant flow diagram for each wave. Each pair of clusters was recruited in 1:1 allocation in one wave each (four waves in total)

In the previous WCQ feasibility study, two areas in Dublin were targeted with populations of 7800 and 8000, respectively. In total, 39 women signed up for the programme, evenly split between the two areas (20 in one area and 19 in the other), and of these, 29 women (74%) were retained at 12 weeks follow-up [15]. Subsequent WCQ programmes (February 2015 to April 2016) have demonstrated this retention rate was maintained at 62% (83/133) (Irish Cancer Society, personal communication). Therefore, based on this existing evidence, if 194 women (97 per arm) are recruited into the study, 120 women are predicted to be in this pilot study by 12 weeks [19]. This will be sufficient to identify potential problems in practicality/feasibility, which have a 5% probability of occurrence [20]. In the previous feasibility study [15], 50% of women who made contact were subsequently recruited to one of two 12-week programmes running concurrently in one district (morning or evening programmes), corresponding to approximately 20 women per programme. If the WCQ programme is run sequentially in 4 districts with 2 concurrent WCQ groups each time (i.e. 8 programmes), then there is potential to recruit up to 160 women during the period January 2018 to June 2019 (18 months), thus exceeding the 97 required. However, the unknown parameters, which need to be estimated from this pilot trial, are the retention rates for the usual care districts, predicted to have much lower rates. Data will also be used to estimate the intra-cluster correlation (ICC), and this data together with recruitment and attrition rates will be used to inform the sample size for the DT. A flow diagram of the study design is attached (Fig. 1). It will be conducted in keeping with the CONSORT statement extended for reporting cluster RCTs [21].

### Criteria for withdrawal

We define ‘withdrawal of consent’ as the participant’s voluntary termination of informed consent to participate which they can give at any point during the conduct of the study. All potentially eligible participants will be fully informed about the study procedures by the participant information leaflet which will state that participation is voluntary and they are free to withdraw at any time without giving a reason. We will retain participants’ data if they withdraw from the study, unless the participant specifically requests withdrawal of their data. All side effects will be reported to the Principal Investigator (PI). Minor side effects will not be regarded as criteria for withdrawal, such as skin irritation, sleep disturbance, and headache or nausea.

### Expressions of interest and informed consent

The LAGs will propose and contact relevant stakeholders to promote the study 12 weeks in advance of programme delivery in their selected pair of districts. Information briefing sessions will be organised by ICS in the community. Each member of the LAG will be provided with promotional material for distribution and will be asked to promote the study through their local channels. Local press releases will be issued. LAG members will be provided with leaflets and posters which will be displayed in local pharmacies, GP surgeries, shops, and dentist and community centres and disseminated outside schools. LAG members will share posts on their organisations’ social media platforms and through their statutory and community contacts. The RA will assist in the promotion of the study through information stands at community events and at local facilities, e.g. creches. The PI will contact the local primary care centres directly and use radio to promote recruitment.

Six weeks in advance of programme delivery, a live project webpage will provide information on the research study and on the districts open for recruitment. Potential participants may express interest in taking part in the study via (i) self-registration through the project website, by telephone or by email, or (ii) referral by their general practitioner, local pharmacist, public health nurse, or community worker who will complete the online registration form on their behalf.

All expressions of interests received will be screened against the inclusion and exclusion criteria. Potentially eligible participants will receive a project information pack containing the participant information leaflet and consent form. The RA will then meet with eligible participants who have had at least 24 h to consider participation to obtain full explicit written consent including consent for salivary sampling. Baseline demographic data will be obtained at time of consent. Consent of recruited women per cluster and across each cluster pair will be obtained prior to breaking the randomisation code; hence, the RA will be blinded to the allocation (Fig. 1).

### Randomisation and allocation concealment

Four matched pairs of districts (eight clusters) will be randomised with each matched pair of clusters randomised to intervention or controls in a 1:1 allocation ratio. The randomisation will follow a pragmatic approach whereby paired districts ready for the intervention will be randomised first, followed by the next available matched pair; this accounts for temporal changes and will provide a balanced design.

The randomisation will be conducted remotely by the Wellcome Trust Clinical Research Facility (CRF) independent of the Trial Management Team, who will develop a standard operating procedure for this process. The code for each matched pair of districts will be concealed and securely stored by the Wellcome CRF at St. James’s Hospital. It will be revealed to the WCQ2 trial team once sufficient numbers have been recruited (or by agreement with the Trial Management Group (TMG) if recruitment is insufficient prior to intervention delivery). Practitioners will be informed of the area in which they will deliver their programme by the research team, and participants will be informed of their allocation by the practitioners. Figure 1 displays an overview of cluster randomisation process.

#### WCQ2 training

Community workers who are identified by the LAG as being interested in delivering the WCQ2 intervention will be trained as facilitators. These potential facilitators may be ex-smokers themselves. The WCQ training programme incorporates the findings of the original WCQ development study [15] and the National Practice Standard in Smoking Cessation [9]. It is a 3-day training programme, co-designed and delivered by HSE specialist trainers and Irish Cancer Society officers. The content includes brief intervention and motivational interviewing techniques in smoking cessation. On successful completion, new CFs are provided with a Community Facilitators Resource Pack [22] containing recommended session plans, resources, and monitoring and feedback tools for the 12-week programme. Within 6 months of the start of programme delivery, CFs are also required to complete an 8–10-h online accredited National Centre for Smoking Cessation Training (NCSCT) programme. Ongoing mentoring and support is provided by each of the partners, and refresher training is provided by the Irish Cancer Society on an annual basis.

All practitioners and researchers involved in our study will be trained in Good Clinical Practice Guidelines.

#### Intervention delivery

Participants randomised to the intervention will receive 12 weekly behavioural support group sessions in a community setting, e.g. resource centre, and NRT dispensed by the community pharmacists. The group sessions are designed to enhance positive social support systems among peers. NRT is made available without cost to all intervention participants.

Each programme will be delivered in a local community facility by two trained CFs. Each session will last approximately 90 min. The CFs will make proactive personal contact both during and between sessions with participants using their own language and cultural style, and provide an opportunity to share testimonials and personal experiences. They will promote social support with delivery of emotional support (e.g. through demonstrating empathetic listening) among peers and will also carry out regular carbon monoxide (CO) monitoring.

#### Control arm

Participants randomised to the control arm will receive a face-to-face individual smoking cessation programme offered by the HSE for men and women delivered by a Smoking Cessation Officer in a community setting [9]. This was chosen as a more suitable control intervention rather than the more readily accessible and widespread telephone and online service due to the difficulty in obtaining data on these services and similarity to the WCQ in terms of providing a face-to-face service. However, it is recognised that only a minority of women have access to this ‘Rolls Royce’ service due to lack of staff availability for service provision.

This programme encompasses, on average, 6–7 individual contacts offered in a primary care centre or hospital outpatient clinic. Sessions vary in duration and mode of delivery; however, the first of these is delivered face-to face (average session length between 30 and 45 min). Subsequent visits occur weekly or every 2 weeks and may be conducted by telephone.

Components of the behavioural support elements of the HSE programme include reinforcing motivation to quit and setting a quit date, building a repertoire of client coping strategies, providing information on the nature of tobacco addiction and withdrawal, undertaking regular carbon monoxide checks and giving feedback on progress, and planning ongoing coping mechanisms and support. Clients are informed about NRT and other smoking cessation pharmacotherapies and are advised to consult their general practitioner on these. HSE clients will obtain these free of charge if they are eligible for free General Medical Services (GMS).

#### HSE standard tobacco cessation training

HSE Smoking Cessation Officers are trained to offer behavioural support and smoking cessation advice. The intensive 2-day training they receive follows the National Standard for Tobacco Cessation Support Programme manual (2013). It includes face-to-face training and online training and assessment. Most HSE Smoking Cessation Officers have clinical backgrounds, e.g. nursing, with some psychotherapy training.

### Outcomes

#### Primary outcome

The primary study outcome is assessment of whether the recruitment target of 8 districts (clusters) and 194 women is achievable within 18 months of programme start. The recruitment rate will be estimated for each wave. It will be defined as the number of women who consent to participate (the numerator), divided by the number of eligible women who have registered with the programme.

The four matched pairs of disadvantaged geographical districts (clusters) will be based on the number of eligible women within in each matched pair of districts (approx. 16,000–20,000). Each pair will be randomised to intervention or to usual care. Subsequent recruitment of 24–25 women who consent to participate in each of the eight districts (97 in each trial arm) will occur during four 12-week periods. The percentage of eligible women needed to plan a DT will be estimated.

Eligibility rates will be established from registration details entered by either the participant or a person acting on their behalf (e.g. GP) on the WCQ2 webpage. Contact details of potential participants will be recorded at the point of expression of interest. Dropout rates will be calculated between time of confirmation of eligibility and time of consent. Number of consented women will be recorded prior to randomisation.

#### Secondary outcomes

The secondary outcomes are as follows:

(i) Retention and data completion rates in each trial arm at 12 weeks and 6 months post-quit date (set in week 2) as a follow-up time point for a future DT
(ii) Proportion who are continuously absent from smoking (as per Russell’s Standard) [23], at 12 weeks (primary outcome for a future DT) and at 6 months after their quit date, corroborated by salivary cotinine and anabasine
(iii) Proportion of enrolled smokers who engage with smoking cessation services in each trial arm (engagement is defined as arrival for a first face-to-face appointment with a smoking cessation adviser and having set a quit date)
(iv) Number of sessions attended in each trial arm
(v) Proportion in each arm who report improvement in health status measured at baseline (week 1) and 12 weeks and 6 months

Data collection will be carried out at week 1, at week 12, and at 6 months post-quit date by the RA via a detailed face-to face questionnaire. Socio-demographic characteristics, smoking behaviour, quality of life (measured by the Short Form-12, Version 2 survey [24]), and health status will be recorded at week 1. Retention and data completion rates, changes in smoking behaviour, and health status will be collected at weeks 6 and 12. Salivary cotinine ± anabasine to biochemically validate self-reported smoking cessation will be recorded at week 12 and 6/12. Attendance sheets will be provided by service delivery providers. A thank you payment (€20 shopping voucher) will be provided at 12 weeks and at 6-month follow-up to encourage continued participation.

### Process evaluation

The aim of the process evaluation is to test the robustness of trial design with respect to context for delivery of the intervention, implementation processes, and strategies from which to optimise trial methodology for progression to a full pragmatic RCT. It will be informed by the Medical Research Council guidance for process evaluation of complex interventions [25] and will use quantitative and qualitative methods to address the research aims. The domains for the process evaluation will be guided by the constructs of the PRECIS-2 tool for assessment of applicability of trial design [26].

The following outcomes will be assessed:

(i) Acceptability of the intervention form and content by participants and trial processes by participants and practitioners involved in service delivery
(ii) Assessment of fidelity (the extent to which the intervention delivered as intended)
(iii) Assessment of contextual factors affecting recruitment and retention
(iv) Assessment of contextual factors including barriers and facilitators to implementation

Acceptability of measures and the experience of being part of a trial will be assessed quantitatively using the Acceptability of Intervention Measure (AIM), Intervention Appropriateness Measure (IAM), and Feasibility of Intervention Measure (FIM) with CFs [27], and qualitatively by semi-structured interviews with participants and with CFs once programme delivery is complete.

The interviews will be conducted using an interview guide. The focus will be on the experiences of those in receipt of the programme and those involved in programme delivery, and of being involved in a research trial. All women participating in the intervention will be invited for interview or will be purposefully sampled depending on numbers [28]. The sampling procedure will target specifically those women who were good attenders, a mix of quitters and continued smokers who completed the 12-week questionnaire comprehensively beforehand (information-rich cases) as a basis to explore the research questions more fully. Data collection will continue until saturation [29]; however, a minimum of 20 qualitative interviews will be conducted with participants. All CFs from each wave of programme delivery will be interviewed jointly, where possible.

Observational field notes will be completed to assist in the assessment of the validity of WCQ2 programme delivery [30]. A log of attendance and any adverse effects experienced by participants will be kept by the CF. Fidelity to training will be carried out through direct observation of training. Fidelity to intervention delivery will be determined using specifically designed checklists and diaries completed by the CFs in tandem once each session is delivered. The checklist will be based upon the delivery of session content determined by an intervention manual [22]. The diary will serve as a reflective tool for the CFs and for the research team to determine any deviations or adaptations in programme delivery. Confidentiality will be assured, as participants will be identified only by their role and will be provided with a pseudonym. Through the combination of diary and checklist, an assessment will be made of whether sessions are delivered as planned, or any adaptations made, in terms of content, frequency, duration, and coverage [31].

Reporting of the study methods will follow published standards for undertaking and reporting qualitative research (COREQ) [32].

The PRECIS-2 is a tool to assist trialists determine the impact of their design decisions on the applicability of an intervention in usual care [26]. The trial will be assessed against the nine domains of the PRECIS-2 wheel: *eligibility*, *recruitment*, *setting*, *organisation*, *flexibility (delivery)*, *flexibility (adherence)*, *follow-up*, *primary outcome*, and *primary analysis.* Each domain of the PRECIS-2 tool will be scored 1 to 5 to indicate how close to usual care the various design aspects of the trial are (5 usual care—pragmatic, and 1 ideal world—explanatory). The tool’s nine domains will be rated and scored by the trial team during and at the end of the intervention.

### Study timelines

Table 1 displays a summary of important time points in the feasibility study. 
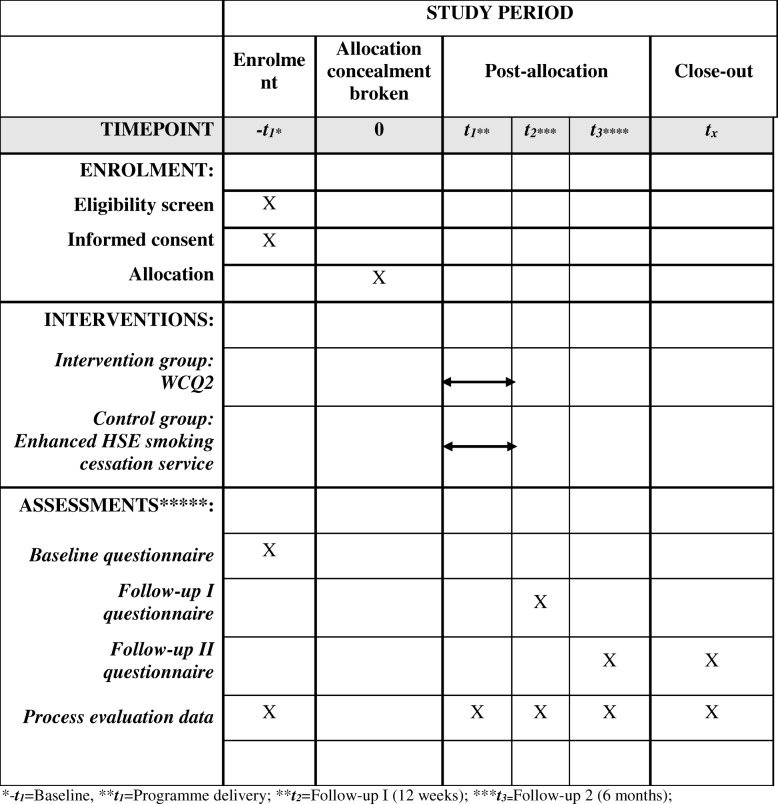

**Table 1:**
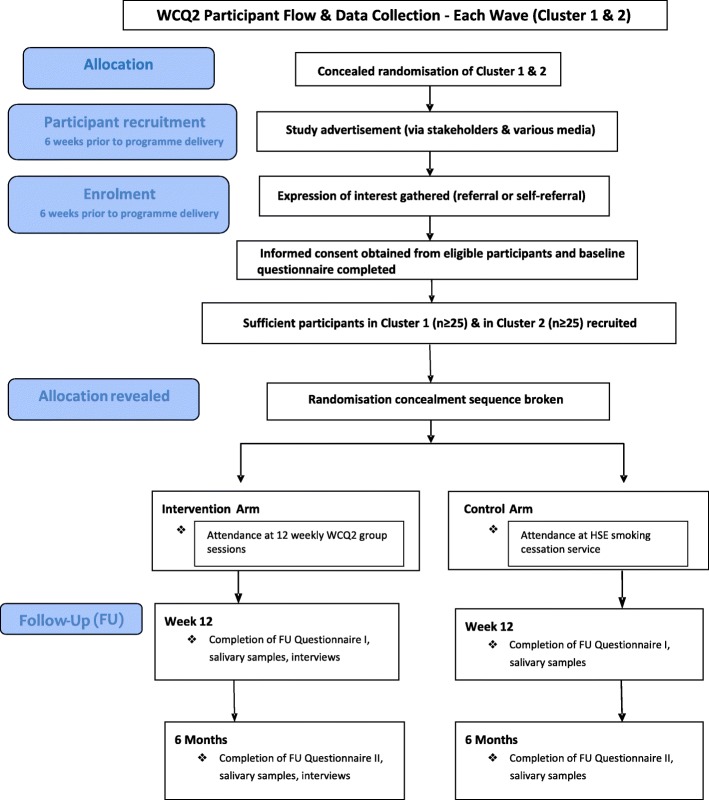
Schedule of enrolment, intervention, and data collection: summary of key trial time points

*−*t*_*1*_ = baseline, **t_*1*_ = programme delivery, ***t_*2*_ = follow-up 1 (12 weeks), ****t_*3*_ *=* follow-up 2 (6 months)

### Data management

All personal participant information will be collected in a separate password-protected file held by the RA. Each participant will be allocated an identification code stored separate from participant responses. The RA will enter the de-identified participant responses in a purpose-built database. A codebook for the database will be developed. A sample of at least 20% percent of data will be checked by the Research Fellow. If the initial check produces an error rate above 1%, a full re-entry of data will be required. If the error rate is less than 1%, the Research Fellow will amend any errors in the final data sheet [33]. The de-identified data will be analysed by the trial team and stored securely for 10 years after which they will be destroyed. Qualitative interview audio files will be recorded on dictaphones and immediately transferred to a password-protected computer. Audio recordings will be sent to a transcriber who will sign a confidentiality agreement and transcribe the recordings verbatim. Transcripts will be stored on a password-protected computer. Salivary samples will be sent to a laboratory in the UK for analysis following the procedures set out in a salivary sample protocol. Only the trial team will have access to the trial data.

### Data analysis

The pilot data will be analysed on an intention-to-treat basis and will be mainly descriptive. A per protocol analysis will also be carried out. A separate statistical analysis plan will be produced. In brief, descriptive analyses will summarise baseline characteristics of women in each trial arm, eligibility rates, recruitment rates, participation, and attrition rates in preparation for a full trial. Subgroup analysis by GMS eligibility will be carried out. Elements of the RE-AIM (Reach, Effectiveness, Adoption, Implementation, and Maintenance) evaluation tool will provide a suitable framework to structure this analysis [34]. As this study is not powered to detect significant differences between groups, formal significance testing will not be carried out. The future trial primary outcome will be the comparison of the proportions of women between groups who self-report having quit smoking, corroborated by laboratory testing such as salivary cotinine at end of programme (12 weeks). The number of participants (%) experiencing cessation will be summarised between groups. The mean change scores (with 95% CIs) between groups on a range of questionnaires will be estimated, to assess whether these were in the expected direction of effect. An assessment of normality will be made on questionnaire scores, and parametric (means and SDs) and/or non-parametric estimates and variability (median/IQR) will be reported at baseline, 12 weeks, and 6 months. Descriptive analysis of secondary outcomes will summarise means and standard deviations for continuous outcome measures or numbers (%) for categorical data at baseline, 12 weeks, and 6 months.

Thematic analysis will be used to analyse qualitative data [35, 36]. Three members of the research team will independently read the transcripts and the diaries. Rigorous line-by-line coding will be applied, with a focus on experiential claims and concerns [37]. Coding will be carried out manually initially and a coding frame developed in Nvivo [37]. Patterns in the data will be clustered into a thematic structure to identify and categorise major themes and sub-themes. Data saturation will be achieved as conceptualised by inductive thematic saturation within the analyses, in relation to the (non-) emergence of new codes or themes [29]. Themes and sub-themes will be reviewed and refined to ensure a coherent pattern and to recode if necessary. Any differences in interpretation will be resolved through discussion. A fourth researcher with qualitative expertise, not involved in the trial, will review the coding frame and apply it to a subset of approximately 10% of the transcripts and two of the four diaries to reduce bias and ascertain the validity of the coding frame as an analytical tool [38]. A kappa coefficient will be calculated for inter-rater reliability. Fidelity to the intervention delivery will be analysed by the number and percentage of self-reported activities completed within each session. Completeness of session content will be noted within individual sessions and across the programme. Differences will be noted for completeness of core and optional session content.

Triangulation of the quantitative and qualitative processes will examine the interaction between context (e.g. group setting, recruitment strategy) and implemented strategies (e.g. training, lay advocate/statutory worker delivery) which led to certain outcomes (e.g. participant engagement, quitting /cutting down, participant satisfaction). The Consolidated Framework for Implementation Research (CIFR) which provides a menu of validated constructs that have been associated with effective implementation will provide a suitable theoretical framework to structure this analysis [39].

#### Progression criteria

This pilot trial will inform the development of a future full DT to assess clinically meaningful and statistically significant outcomes which may include an economic evaluation to determine cost-effectiveness, which is not part of the feasibility study. The ADePT decision aid will be used alongside PRECIS-2 to aid the decision-making process around progression to the full DT [40]. ADePT provides an algorithm which enables decisions to be made in a structured way which determine whether problems encountered were trial, or real world problems, and how they can be overcome for a future trial.

### Dissemination

Once trial results are available, the trial team will prepare accessible briefings for the key organisations interested in the research. These briefings will highlight results and key lessons learned during the conduct of the trial and inform the design of any future study. A lay summary of results will also be prepared for trial participants, developed with Public and Patient Involvement (PPI) representatives. Final pilot trial results will be available via a report to the funder, submission of articles to peer-reviewed journals, an updated summary and briefing papers for professionals and the public, presentations at relevant meetings and conferences, and a summary report on the project website.

## Discussion

The WCQ2 builds on the previously evaluated tailored smoking cessation programme offered by the Irish Cancer Society for women living in deprived areas of Ireland [15]. WCQ2 aims to now pilot this intervention to inform the design of a future potential DT. It is also important to describe not only the intervention but also the comparator, usual care. Usual care varies across Ireland, and the face-to-face service is not universally available. The funding and ongoing resourcing of this service will have implications for the implementation of a future trial.

As with any pilot trial, the main challenges are likely to be recruitment and retention. The trial recruitment processes, informed consent and randomisation, may act as barriers to rapid uptake of the WCQ and HSE programmes. Other challenges include the seasonal nature of attempts to stop smoking (less likely to occur during the summer and holiday periods, and thus, women may be less motivated to participate in cessation programmes). Although our trial is described as pragmatic, there is a tension between the need to adhere to a rigorous clinical trial design (the explanatory-pragmatic continuum) while maintaining study conditions as closely as possible to usual practice [41]. Our use of the PRECIS-2 tool [26] will analyse the extent to which WCQ2 is a pragmatic trial, so as to optimise the trial’s applicability to the needs of intended stakeholders in the full DT. The embedded process evaluation will inform an assessment of the acceptability of the intervention and process of conducting an RCT in disadvantaged community settings and facilitate the design of a future DT or alternative trial design, taking into account population size and anticipated problems of low recruitment, attrition, and validated outcome measures.

Conducting this pilot study as described will enable an assessment of the feasibility of addressing the primary and secondary outcomes of a future DT of a smoking cessation intervention tailored to disadvantaged women which is delivered by trained peers in their local community. It will provide insights into how to optimise recruitment strategies and data collection and into the acceptability of trial measures including randomisation. It will provide transferable learning on the systems and implementation strategies needed to support effective design of future pragmatic community-based trials which address health promotion interventions for women in disadvantaged communities.

## Supplementary information

**Additional file 1.** SPIRIT checklist.

## Data Availability

Not applicable.

## Abbreviations

AIM: Acceptability of Intervention Measure
CI: Confidence interval
CIFR: Consolidated Framework for Implementation Research
CO: Carbon monoxide
CONSORT: Consolidated Standards of Reporting Trials
DT: Definitive trial
FIM: Feasibility of Intervention Measure
GMS: General Medical Services scheme
HSE: Health Service Executive
IAM: Intervention Appropriateness Measure
ICC: Intra-cluster correlation coefficient
IQR: Interquartile range
LAGs: Local Advisory Groups
NRT: Nicotine Replacement Therapy
RA: Research Assistant
RCT: Randomised controlled trial
RE-AIM: Reach, Effectiveness, Adoption, Implementation, Maintenance
SD: Standard deviation
SOP: Standard operating procedure
WCQ: We Can Quit
Wellcome CRF: Wellcome Clinical Research Facility
WHO: World Health Organisation
